# Prenatal exposure to air pollution is associated with structural changes in the neonatal brain

**DOI:** 10.1016/j.envint.2023.107921

**Published:** 2023-04

**Authors:** Brendan Bos, Ben Barratt, Dafnis Batalle, Oliver Gale-Grant, Emer J. Hughes, Sean Beevers, Lucilio Cordero-Grande, Anthony N. Price, Jana Hutter, Joseph V. Hajnal, Frank J. Kelly, A. David Edwards, Serena J. Counsell

**Affiliations:** a MRC Centre for Environment and Health, Imperial College London, UK; b Centre for the Developing Brain, School of Biomedical Engineering and Imaging Sciences, King’s College London, London SE1 7EH, UK; c Department of Forensic and Neurodevelopmental Science, Institute of Psychiatry, Psychology & Neuroscience, King’s College London, UK; d Biomedical Image Technologies, ETSI Telecomunicación, Universidad Politécnica de Madrid and CIBER-BBN, Madrid, Spain

**Keywords:** Prenatal air pollution exposure, Neonate, Brain, MRI

## Abstract

**Background:**

Prenatal exposure to air pollution is associated with adverse neurologic consequences in childhood. However, the relationship between in utero exposure to air pollution and neonatal brain development is unclear.

**Methods:**

We modelled maternal exposure to nitrogen dioxide (NO_2_) and particulate matter (PM_2.5_ and PM_10_) at postcode level between date of conception to date of birth and studied the effect of prenatal air pollution exposure on neonatal brain morphology in 469 (207 male) healthy neonates, with gestational age of ≥36 weeks. Infants underwent MR neuroimaging at 3 Tesla at 41.29 (36.71–45.14) weeks post-menstrual age (PMA) as part of the developing human connectome project (dHCP). Single pollutant linear regression and canonical correlation analysis (CCA) were performed to assess the relationship between air pollution and brain morphology, adjusting for confounders and correcting for false discovery rate.

**Results:**

Higher exposure to PM_10_ and lower exposure to NO_2_ was strongly canonically correlated to a larger relative ventricular volume, and moderately associated with larger relative size of the cerebellum. Modest associations were detected with higher exposure to PM_10_ and lower exposure to NO_2_ and smaller relative cortical grey matter and amygdala and hippocampus, and larger relaive brainstem and extracerebral CSF volume. No associations were found with white matter or deep grey nuclei volume.

**Conclusions:**

Our findings show that prenatal exposure to air pollution is associated with altered brain morphometry in the neonatal period, albeit with opposing results for NO_2_ and PM_10_. This finding provides further evidence that reducing levels of maternal exposure to particulate matter during pregnancy should be a public health priority and highlights the importance of understanding the impacts of air pollution on this critical development window.

## Highlights

• Examined effects of prenatal exposure to air pollution on neonatal brain structure.
• Large study in healthy term born neonates.
• Prenatal air pollution exposure was associated with altered brain morphology.

## 1 Introduction

Exposure to air pollution is associated with impaired brain health and cognitive ability (Russ et al., 2019). Higher exposure throughout the life course has been linked to increased risk of cognitive decline (Clifford et al., 2016). The developing brain is particularly susceptible to the effects of environmental exposures due to its rapid development, leading to vulnerability in key maturation processes (Grandjean and Landrigan, 2014). Exposure to a wide range of pollutants in childhood has been associated with developmental delay (Clifford et al., 2016). In addition, maternal exposure during pregnancy may adversely impact brain development, disrupting neurodevelopment and increasing the risk of cognitive dysfunction (Jedrychowski et al., 2015, Volk et al., 2021).

Particulate matter (PM) and traffic-related air pollution (TRAP) exposure can lead to microglial activation, inflammation, mitochondrial dysfunction, and oxidative stress in the brain (Morris et al., 2021, Gómez-Budia et al., 2020). Similarly, maternal exposure may lead to systemic inflammation, which is harmful to fetal neurodevelopment (Nachman Rebecca et al. , 2016, Yan et al., 2019). Black carbon (BC) and other particles associated with traffic emissions have been found in the placenta (Bové et al., 2019, Liu et al., 2021), and could be harmful to placental function. Impaired placental function is associated with abnormal brain development in animal models (Batalle et al., 2014) and is a candidate mechanism for altered brain development associated with air pollution (Luyten et al., 2018). BC particles were also detected in first and second trimester fetal brain tissues and could be responsible for adverse effects on brain development (Bongaerts et al., 2022).

Several animal studies have examined the relationship between prenatal exposure to air pollution and brain morphology in early life, however, these provide conflicting results. In mice, increased exposure to PM_2.5_ or ultrafine particles was associated with reduced corpus callosum volume (di Domenico et al., 2020, Allen et al., 2017), increased corpus callosum and reduced hippocampal volume (Klocke et al., 2017), ventriculomegaly in females (Klocke et al., 2018a) and males (Allen et al., 2017). Increased exposure to diesel exhaust particles was associated with increased cortical volume in males (Bolton et al., 2017). In female rats, decreased lateral ventricular volume was observed on magnetic resonance imaging (MRI) following increased exposure to TRAP (Patten et al., 2020).

MRI allows non-invasive in-vivo assessment of brain structure. Several studies have examined the impact of prenatal exposure to air pollution on brain morphology in school age children using MRI, and results have shown mostly adverse findings. Prenatal exposure to PM_2.5_ was associated with thinner cortex in children aged 6 to 10 years old (Guxens et al., 2018), but not with global brain volumes. Prenatal exposure to polycyclic aromatic hydrocarbons (PAH) was associated with reduced white matter surface area in children aged 7 to 9 years old (Peterson et al., 2015). Further, a study which considered the effect of prenatal exposures to a wide range of pollutants in children aged 9 to 12 years old while also adjusting for postnatal exposures detected associations between increased exposure and larger amygdala, cerebellum, putamen and pallidum volume, smaller corpus callosum and hippocampal volume (Lubczyńska et al., 2021). In addition, a recent study associated prenatal exposures to PM_2.5_ and PAH with smaller white matter (WM) volumes (Peterson et al., 2022). However, none of these studies undertook neonatal brain MRI.

In this study we aimed to examine the relationship between in utero exposure to air pollutants and neonatal brain development using brain MRI data acquired shortly after birth. We hypothesised that prenatal exposure to air pollution is associated with altered neonatal brain morphology. We assessed the impact of prenatal exposure to three ubiquitous air pollutants, PM_10_, PM_2.5_, and nitrogen dioxide (NO_2_), which have previously been linked to altered brain development in older children.

## 2 Methods

### 2.1 Subjects

This study is based on a sample of neonates participating in the Developing Human Connectome Project (dHCP, http://www.developingconnectome.org/), a cohort recruited in Greater London, UK. Research Ethics Committee approval was granted for this project (14/LO/1169) and written informed consent was obtained from parents. The principal inclusion criteria of the neonatal dHCP were infants born between 23- and 44-weeks gestational age, estimated from the mothers last menstrual period and confirmed where possible by early ultrasound scanning (https://www.developingconnectome.org/study-inclusion-and-exclusion-criteria/). Infants were recruited at St Thomas’ Hospital, London and imaged at the Evelina Newborn Imaging Centre, Centre for the Developing Brain, King’s College London, United Kingdom. The subjects in this study were born between the years 2015 to 2020 and were scanned in the neonatal period. 782 infants were included in the dHCP project at the time of this study. Infants were excluded if their mothers smoked during pregnancy (n = 16), gestational age (GA) at birth was 36 weeks or less (n = 94), or 44 weeks or more (n = 0), or there was evidence of major focal lesions on MRI (n = 29). In cases of multiple pregnancies (n = 46), only one infant of the twin pair was included. In addition, 128 infants were excluded as the mother’s residential address was outside of the air pollution model domain. The final sample consisted of 469 infants. Index of multiple deprivation (IMD) was calculated from postcode at birth for all infants using an online tool (https://tools.npeu.ox.ac.uk/imd/). IMD is a composite measure of socioeconomic status in England encompassing factors such as income, employment, education, health and crime and will be expressed in quintiles throughout this paper (https://opendatacommunities.org/data/societal-wellbeing/imd/indices).

### 2.2 MRI acquisition

MRI data from each neonate was acquired on a Phillips 3-Tesla Achieva system (Philips Medical Systems, Best, The Netherlands). All infants were scanned during natural sleep without sedation using a dedicated protocol as previously described by our group (Hughes et al., 2017), including a bespoke transport system, positioning device and a customized 32-channel receive coil with a custom-made acoustic hood. All scans were supervised by a neonatal nurse and/or paediatrician who monitored heart rate, oxygen saturation and temperature throughout the scan.

T2-weighted, T1-weighted, and diffusion-weighted MR images were acquired using the dHCP protocol optimized for neonatal scanning (The Developing Human Connectome Project, (2019) https://www.developingconnectome.org/; (Hughes et al., 2017, Hutter et al., 2018, Edwards et al., 2022)). T2-weighted images were used in these analyses and were obtained using a turbo spin echo (TSE) sequence, acquired in two stacks of 2D slices (in sagittal and axial planes), using parameters: TR = 12 s, TE = 156 ms, SENSE factor 2.11 (axial) and 2.58 (sagittal) with overlapping slices (resolution 0.8 × 0.8 × 1.6 mm^3^). All images were reviewed by a perinatal neuroradiologist.

### 2.3 Image processing

Motion-correction and slice-to-volume image reconstruction were carried out retrospectively using a dedicated algorithm to obtain 0.8 mm^3^ isotropic T2-weighted images (Cordero-Grande et al., 2018, Kuklisova-Murgasova et al., 2012). These were segmented into the following tissue types; white matter, cortical grey matter (cGM), ventricles, extracerebral cerebrospinal fluid (CSF), cerebellum, hippocampus & amygdala, deep grey matter, brainstem using a multi-structure expectation–maximization-based segmentation technique in a neonatal-specific automated pipeline described previously (Makropoulos et al., 2016, Makropoulos et al., 2014, Makropoulos et al., 2018).

### 2.4 Air pollution modelling

Maternal air pollution exposure was modelled using the London Air Pollution Toolkit (Beevers et al., 2012). The Air Pollution Toolkit uses a combined modelling-measurement approach and a kernel modelling technique to describe pollution dispersion. It simulates traffic exhaust emissions based upon hourly traffic flows and speeds, along each of the road links using a London specific vehicle stock and the National Atmospheric Emissions Inventory (NAEI). Emission sources other than road transport were taken from the London Atmospheric Emissions Inventory (LAEI). The toolkit is capable of modelling more than one million individual sources with different source characteristics and has an output grid resolution of 20 × 20 m. The pollution value used to represent a postcode was based on centroids inside the postcode. The location of the centroids depended on each individual postcode’s environment, whether it is fully built up, surrounding road type, and whether there are green spaces inside it. A more detailed description of the Toolkit and its quality assurance and quality control can be found at: https://www.healtheffects.org/system/files/Kelly-LEZ-AppendixB.pdf (Kelly et al., 2011). Temporal adjustment was applied to annual mean model estimates using aggregated measurements from the London Air Quality Network (www.londonair.org.uk) using the method described in Whitehouse *et al* (Whitehouse et al., 2020).

Air pollution exposure was modelled at postcode level. Postcode units in the UK are very small, covering 15 households on average, and there are over 150,000 postcode units in Greater London. We used maternal residence and modelled the time between estimated date of conception and date of birth to obtain an average exposure over the gestational period. We also modelled air pollution exposure for each trimester separately. The modelled pollutants were PM_2.5_, PM_10_, and NO_2_.

#### 2.4.1 Statistical analysis

Data were tested for normality using the Shapiro-wilk test (Shapiro and Wilk, 1965). Statistical analysis was performed using R studio (Version 4.1.2, https://www.r-project.org/). All volumes were adjusted for intracranial volume (ICV) to remove the effect of individual differences in head size and will be referred to as “relative” volumes throughout this paper. The relationship between prenatal pollution exposure and brain morphology was first explored using single pollutant linear regression analysis, adjusting for postmenstrual age (PMA) at scan, gestational age (GA) at birth, sex, and IMD. Results were false discovery rate corrected using Benjamini-Hochberg false discovery rate method (Benjamini and Hochberg, 1995).

In order to include multiple colinear pollution and brain variables in one model we then used canonical correlation analysis (CCA) (using the CCA package in R (https://cran.r-project.org/web/packages/CCA/CCA.pdf, version 1.2.1, (González et al., 2008)). CCA is a multidimensional statistical method that looks for a weighted linear composite for each variate (sets of dependent and independent variables) to maximize the overlap in their distributions (termed a mode), whilst simultaneously addressing multi-collinearity (Krzanowski, 1988).

To account for potential confounding factors relevant to neonatal MRI, all brain volumes included in the analysis were adjusted for PMA at scan, GA at birth, and sex. Further, to account for socioeconomic status, the residuals of pollutant variables were determined and adjusted for IMD as it has been shown to be correlated with pollution exposure (Hajat et al., 2015). For each CCA mode, we used a permutation testing procedure to test the significance of the corresponding canonical correlation as a validation step. To perform this, the rows of both inputs (brain variable and pollution variables) were permuted 1000 times, CCA was re-run and canonical correlations re-computed after each permutation. A mode of the resulting CCA was only considered significant if the Spearman’s *r* was greater than the 95th percentile of random permutation tests (Yu et al., 2019). Significance of the association between modes that passed this test was corrected for multiple comparisons using the Benjamini-Hochberg false discovery rate method (Benjamini and Hochberg, 1995). Finally, the correlation of each original variable to the CCA mode testing for univariate correlation was assessed between each of the brain measures and pollutants using Spearman’s r test.

#### 2.4.2 Secondary analyses

A secondary analysis was performed to examine the impact of trimester specific exposures on neonatal brain morphology. All volumes were adjusted for ICV, PMA at scan, GA at birth, sex, and IMD in a single pollutant linear regression analysis. Further, trimester specific air pollution exposure estimates were adjusted for seasonality due to the disproportionate effect of seasonality on shorter exposure periods. A CCA was then performed for each trimester as outlined above. We examined sex-specific associations between prenatal exposure to air pollution and neonatal brain morphology and investigated the correlations between exposures by trimester using Spearman’s rank correlation (more details can be found in the supplement).

## 3 Results

### 3.1 Study sample

**Fig. 1:**
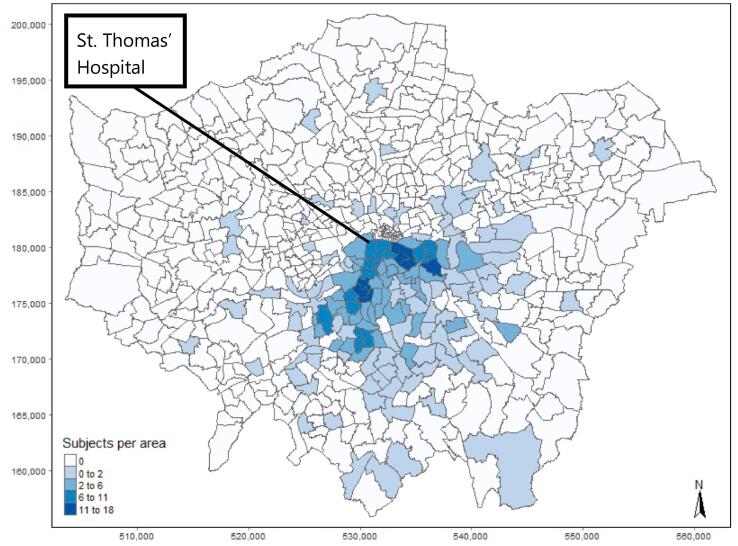
Subject distribution of our study sample taken from the developing human connectome project (dHCP) per lower super output area (LSOA) in Greater London. The subjects were scanned at St. Thomas’ Hospital in central London.

The study sample comprised 469 infants (207 male) born at a median (range) GA of 40.14 (36.14–43.57) weeks and imaged at 41.29 (36.71–45.14) weeks PMA (Table 1). The median IMD quintile for the study group was 4 (1–5). Male subjects were scanned at a median age of 41.43 weeks, whereas female subjects were scanned at a median date of 41.07 weeks (Table 2). Subjects were concentrated around the Boroughs of Southwark and Lambeth, close to where the MRI was performed (Fig. 1). This map shows the subject distribution per Lower Super Output Area (LSOA), not per postcode, to preserve anonymity.

**Table 1:** Distribution of age at MRI, age at birth, sex, and index of multiple deprivation of the study sample from the developing human connectome project (dHCP).

Demographic information	
Age at MRI, weeks (median, range)	41.29 (36.71 – 45.14)
Age at birth, weeks (median, range)	40.14 (36.14 – 43.57)
Sex (n, %)	
Female	262 (55.9%)
Male	207 (44.1%)
Index of multiple deprivation quintile (median, range)	4 (1–5)

**Table 2:** Distribution of age at MRI, age at birth, and index of multiple deprivation of the study sample from the developing human connectome project (dHCP) stratified by sex.

	Male	Female
Age at MRI, weeks (median, range)	41.43 (36.86 – 45.14)	41.07 (36.71 – 44.86)
Age at birth, weeks (median, range)	40.14 (36.29 – 43)	40.14 (36.14 – 43.57)
Index of multiple deprivation quintile (median, range)	4 (1 – 5)	4 (1 – 5)

### 3.2 Pollution exposure

Descriptive statistics of subjects’ pollution exposure are detailed below (Table 3). NO_2_ and PM_10_ were non-normally distributed, summary statistics are therefore presented as median and range. A map of annual mean PM_10_ and NO_2_ concentrations across Greater London in 2016 is included in the supplement (Figs. S1 and S2) to illustrate spatial exposure gradients.

**Table 3:** Descriptive statistics of modelled pollution exposure for each trimester and the whole pregnancy in µg/m^3^ for our study sample taken from the developing human connectome project (dHCP) based in London.

**PM_10_** **levels, μg/m^3^** (median, range)	
1st trimester of pregnancy	23.1 (17.1 – 37.1)
2nd trimester of pregnancy	23.2 (15.6 – 38.5)
3rd trimester of pregnancy	22.8 (11.8 – 39.5)
Whole pregnancy	23.4 (18.2 – 30.5)

**PM_2.5_** **levels, μg/m^3^** (median, range)	
1st trimester of pregnancy	13.4 (9.0 – 24.2)
2nd trimester of pregnancy	13.5 (8.5 – 25.1)
3rd trimester of pregnancy	13.2 (8.9 – 22.5)
Whole pregnancy	13.5 (10.1 – 18.4)

**NO_2_ levels, μg/m^3^** (median, range)	
1st trimester of pregnancy	39.4 (23.3 – 59.0)
2nd trimester of pregnancy	39.6 (20.2 – 71.7)
3rd trimester of pregnancy	38.3 (21.9 – 62.3)
Whole pregnancy	39.7 (24.73 – 58.0)

### 3.3 Brain volumes

Descriptive statistics of subject’s brain volumes are detailed below (Table 4). Total brain volume (TBV) and relative cerebellum were non-normally distributed and so summary statistics are presented as median and range. A segmented image in a neonate at 40 weeks PMA showing the analysed brain regions can be found in the supplement (Fig. S3).

**Table 4:** Median brain volumes for our study sample taken from the developing human connectome project (dHCP) based in London.

Volume	Median	Range
Intracranial Volume (mm^3^)	439,103	273,072 – 664,283
Total Brain Volume (mm^3^)	361,807	223,962 – 532,365
Relative Cortical Grey Matter	0.419	0.365 – 0.496
Relative White Matter	0.422	0.342 – 0.466
Relative Cerebellum	0.07	0.054 – 0.089
Relative Ventricles	0.013	0.007 – 0.047
Relative Deep Grey Nuclei	0.075	0.063 – 0.087
Relative Brainstem	0.018	0.015 – 0.022
Relative Amygdala & Hippocampus	0.007	0.0058 – 0.0088
Relative Extracerebral CSF	0.168	0.112 – 0.253

#### 3.3.1 Single pollutant linear regression results

Table 5 shows the results of the single pollutant linear regression model assessing the relationship between pollution exposure over the entire gestational period and relative brain volumes. None of the associations survived false discovery rate correction. This was also the case when examining exposure per trimester (Tables S1, S2, and S3).

**Table 5:** Single pollutant linear regression results for each brain volume and pollutant.

Brain region (relative)	Pollutant	beta	95% CI	p-value	p-FDR
White Matter	NO_2_	−0.00004	−0.0003, 0.0001	0.68	0.96
	PM_2.5_	−0.0001	−0.0009, 0.0004	0.63	0.96
	PM_10_	−0.0001	−0.0007, 0.0003	0.69	0.96
Cortical Grey Matter	NO_2_	0.00006	−0.0001, 0.0003	0.52	0.96
	PM_2.5_	−0.00005	−0.0005, 0.0008	0.88	0.98
	PM_10_	0.00002	−0.00044, 0.0006	0.94	0.98
Cerebellum	NO_2_	−0.00005	−0.0001, 0.00003	0.27	0.81
	PM_2.5_	0.000002	−0.0003, 0.0002	0.99	0.99
	PM_10_	0.000009	−0.0003, 0.0002	0.94	0.98
Brainstem	NO_2_	−0.00001	−0.00001, 0.00005	0.24	0.81
	PM_2.5_	−0.00004	−0.00002, 0.00009	0.17	0.68
	PM_10_	0.00004	−0.00001, 0.00009	0.07	0.53
Ventricle	NO_2_	−0.00002	−0.0001, 0.00004	0.64	0.96
	PM_2.5_	−0.0001	−0.0001, 0.0003	0.31	0.83
	PM_10_	0.0001	−0.00006, 0.0003	0.09	0.53
Deep Grey Nuclei	NO_2_	−0.00001	−0.00004, 0.00007	0.66	0.96
	PM_2.5_	0.00007	−0.0001, 0.0003	0.48	0.96
	PM_10_	0.00003	−0.0001, 0.0002	0.69	0.96
Extracerebral CSF	NO_2_	0.0003	−0.0001, 0.0006	0.11	0.53
	PM_2.5_	0.001	0.00004, 0.002	0.02	0.24
	PM_10_	0.001	−0.00003, 0.0019	0.02	0.24
Amygdala & Hippocampus	NO_2_	−0.000006	−0.000007, 0.00001	0.76	0.96
	PM_2.5_	−0.000001	−0.00003, 0.00003	0.93	0.98
	PM_10_	−0.000003	−0.00002, 0.00002	0.76	0.96

### 3.4 CCA results

#### 3.4.1 Entire pregnancy

**Fig. 2:**
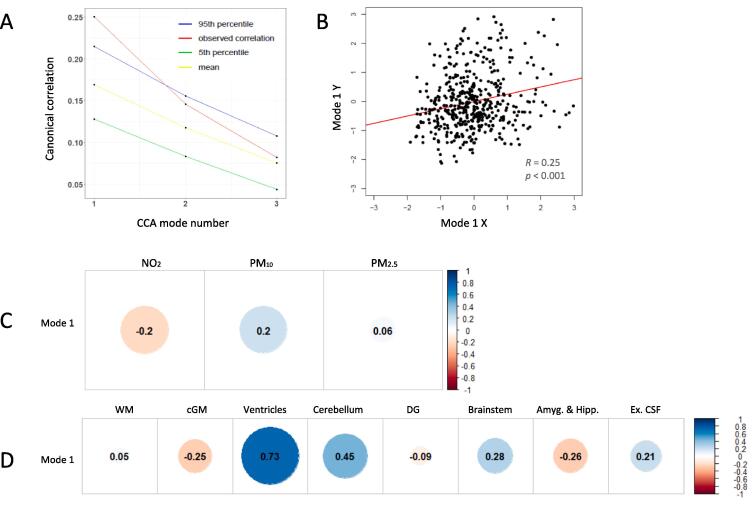
(A) Observed CCA correlations (y-axis), the mean, and the fifth to 95th percentiles of the null distribution of the permuted CCA correlations estimated via permutation testing across the three CCA modes (x-axis). (B) Scatterplot of individual correlations of CCA mode 1. (C) Canonical correlation of the pollutants using Spearman’s rank test, in the following order: 1. NO_2_, 2. PM_10_, 3. PM_2.5_. (D) Canonical correlations of the relative brain volumes using Spearman’s rank test, in the following order: 1. White Matter, 2. Cortical Grey matter, 3. Ventricles, 4. Cerebellum, 5. Deep Grey Nuclei, 6. Brainstem, 7. Amygdala & Hippocampus, 8. Ex. Cerebral CSF.

CCA of exposure to air pollution over the entire gestational period identified three modes. Only mode 1 exceeded the 95th percentile of random permutations (Fig. 2).

Higher PM_10_ concentrations and lower NO_2_ concentrations were strongly canonically correlated to a larger relative size of the ventricles, moderately associated with larger relative size of the cerebellum, and modestly associated with smaller size of cGM, amygdala & hippocampus, and a larger brainstem and extracerebral CSF in the first mode (Table 6, Table 7, Fig. 2). Exposure to PM_2.5_ was not significantly associated with any brain volume measures. All brain region associations with the first mode except white matter and deep grey nuclei survived false discovery rate correction.

**Table 6:** Spearman’s R and R^2^ for each relative region of the brain and the association with the first mode.

Brain Region	Spearman’s R	R^2^	p-value	p-FDR
White Matter	0.055	0.003	0.24	0.24
Cortical Grey Matter	−0.251	0.063	<0.001	<0.001
Ventricles	0.735	0.540	<0.001	<0.001
Cerebellum	0.450	0.202	<0.001	<0.001
Deep Grey Nuclei	−0.087	0.007	0.06	0.07
Brainstem	0.281	0.079	<0.001	<0.001
Amygdala & Hippocampus	−0.261	0.068	<0.001	<0.001
Extracerebral CSF	0.215	0.046	<0.001	<0.001

**Table 7:** Spearman’s R and R^2^ for each pollutant and the association with the first mode.

Pollutant	Spearman’s R	R^2^	p-value	p-FDR
NO_2_	−0.2	0.04	<0.001	<0.001
PM_10_	0.2	0.04	<0.001	<0.001
PM_2.5_	0.06	0.004	0.2	0.2

All pollutants had significant but modest associations with the first mode (Table 5).

#### 3.4.2 Trimesters

CCA of exposure to air pollution over the trimesters identified three modes, however, none of these exceeded the 95th percentile of random permutations.

## 4 Discussion

In this study we assessed the relationship between prenatal exposure to air pollution and brain morphology in the neonatal period using MRI, and to our knowledge this is the first study to do so. Using CCA, we observed associations between air pollution in utero and neonatal brain structure with higher gestational exposure to PM_10_ and lower exposure to NO_2_ related to larger relative ventricular and cerebellar volume, along with modest associations with smaller relative cGM, amygdala and hippocampus volumes, and larger relative volumes of brainstem and extracerebral CSF.

To date, studies assessing prenatal exposure to air pollutants and subsequent brain development provide conflicting results. Lubczynska *et al* found an association between increased exposure to coarse PM (which is a derivative of the PM exposures assessed in this paper, PM_10_ - PM_2.5_) during pregnancy and increased cerebellar size in children age 9–12, and an association between increased exposure to PM_2.5_ and smaller cerebellar volume in their multipollutant model, though this latter finding was not statistically significant in their single-pollutant model (Lubczyńska et al., 2021). A study examining TRAP exposure during the first year of life showed an association with smaller cerebellum in children aged 12 years old (Beckwith et al., 2020). These findings relate to MRI scans taken in childhood, however, and as such are not directly comparable to our data.

We found larger relative ventricle volumes in relation to increased exposure to PM_10_, and decreased exposure to NO_2_. In mice, Allen *et al* detected an association between increased ultrafine particle exposure during pregnancy and male-specific ventriculomegaly. Ventriculomegaly occurred in concert with inflammation and microglial activation, aberrant WM development and hypomyelination in the corpus callosum (Allen et al., 2017). Klocke *et al* also related higher gestational exposure to concentrated ambient particles in mice with ventriculomegaly (Klocke et al., 2017). In our study we observed larger relative size of the ventricles as a result of exposure to higher levels of PM_10_. Ventriculomegaly could reflect an expansion of the ventricles to fill available space due to cell loss and/or altered brain development (Allen et al., 2017, Leviton and Gilles, 1996).

The finding that lower NO_2_ concentrations are related to larger relative ventricle size is unexpected. One other study reported opposing effects of NO_2_ and PM_2.5_, in which the third mode of CCA linked higher concentrations of PM_2.5_ to lower concentrations of NO_2_, and was related to lower area and volumes across the cortex as well as white matter indices in school-aged children (Alnæs et al., 2020). NO_2_ may affect the developing brain in several ways, and prenatal exposure could harm the fetal brain by inducing inflammation, oxidative stress, DNA methylation, and vascular injury (Shang et al., 2020). However, there is some evidence that acute exposure to high concentrations of NO_2_ increases nitrite in the blood stream and lowers blood pressure (Floyd et al., 2020). In this study we modelled chronic ambient exposures, which tend to be lower than acute exposures via domestic gas appliances as used in Floyd *et al*, and there is no proven link between long-term exposure to NO_2_ and lower blood pressure.

Higher gestational exposure to PM_10_ and lower exposure to NO_2_ was related to larger relative cerebellar volume in our study. The mechanisms relating increased PM exposure in utero and increased cerebellar size are not clear. In rat brains, direct exposure of the cerebellum to PM_2.5_ in solution was shown to cause oxidative stress (Fagundes et al., 2015), and inhaled diesel-extracted particles led to oxidative stress and inflammation in the cerebellum in mice (Kim et al., 2018). Again, however, comparison is difficult as these studies were conducted during different time periods, namely in adult and adolescent animals, respectively. Klocke *et al* examined young mice and found that gestational exposure to ambient PM_2.5_ was related to hypermyelination in the cerebellum of male mice (Klocke et al., 2018b). This last finding points to accelerated maturation as a result of high gestational exposure to air pollutants, and as theorized by Klocke *et al*, may be a response to injury.

Air pollution exposure explained a small amount of variance in cGM, amygdala & hippocampus, brainstem, and extracerebral CSF volumes. Higher gestational exposure to PM_10_ and lower exposure to NO_2_ was related to smaller relative cGM and amygdala and hippocampus volumes, and larger relative brainstem and extracerebral CSF volumes. Some of these findings are comparable to previous studies in children. Guxens et al observed that higher pregnancy and childhood exposure to multiple air pollutants was related to smaller hippocampus and larger amygdala in 12-year-olds with no associations found for cGM (Guxens et al., 2022). Beckwith et al also found reduced cGM volume in children aged 12 years old after childhood exposure to traffic-related air pollution (Beckwith et al., 2020).

In mice, decreased volume of the hippocampus was associated with prenatal exposure to ultra-fine particles (Klocke et al., 2017). Woodward et al also found impaired neurogenesis in the hippocampus in adult rats exposed prenatally to TRAP (Woodward et al., 2018). Impaired neurogenesis as a consequence of higher exposure to PM_10_ may contribute to the smaller relative cGM, hippocampal and amygdala volumes we observe in our study.

There have been few studies analysing brain volumes in healthy term infants in the neonatal period (Gale-Grant et al., 2022). However, in infants born preterm, increased ventricular volume (Liverani et al., 2023) and decreased cGM volume (Liverani et al., 2023, Pagnozzi et al., 2023) in the neonatal period were associated with lower outcome scores at 2 years. In addition, Liverani *et al*, demonstrated a relationship between amygdala volumes in the neonatal period and behavioural scores in 5-year-old children born preterm.

Air pollution concentrations modelled for our London-based cohort are comparable to those found in other studies examining pregnancy cohorts in European cities (Guxens et al., 2022). Pregnancy cohorts studied in low to middle income countries have found much higher exposures (Wylie Blair et al., 2017, Zhou et al., 2022), whereas pregnancy cohorts in north America have generally found lower exposures to e.g. PM_2.5_ (Zhang et al., 2022, Zhang et al., 2018). Thus, our findings are comparable to other European cohorts. However, to our knowledge no other studies have examined neonatal brain MRI in relation to prenatal air pollution exposure.

We did not observe any associations between individual trimester exposure and brain volumes, nor was prenatal exposure to PM_2.5_ significantly associated with any of the brain volumes studied. Further, we did not observe any significant associations between exposure over the entire gestational period and WM or deep grey nuclei volume, despite previous studies showing associations in children aged 6–14 years old (Pujol et al., 2016, Mortamais et al., 2017, Cserbik et al., 2020, Lubczyńska et al., 2021, Peterson et al., 2022, Peterson et al., 2015). It is possible that these relationships become apparent only in later childhood. When we divided the participants into male and female, we did not find any sex-specific associations between prenatal exposure to air pollution and brain volumes. This may be due to reduced statistical power resulting from dividing the study sample into male and female subgroups. Including sex as a term in the CCA model was not possible, as categorical variables cannot be included in CCA.

Strengths and limitations.

A strength of this study is the large sample size for a neonatal cohort, which is comparable to cohorts in studies with MRI taken in childhood. Further, bespoke imaging methods were used to obtain state of the art neonatal imaging, and air pollution data was modelled using a validated high-quality ambient air pollution model. Finally, the statistical method used allowed incorporation of three ubiquitous yet highly correlated pollutants.

Our study has some limitations. Firstly, IMD does not include individual level variables of socioeconomic status, as it is modelled at postcode level. It therefore does not fully adjust for factors that have been shown to influence fetal brain development, such as maternal education. Ambient air pollution concentrations were also modelled on postcode level. There may be heterogeneity within postcodes, even if postcodes in the UK are often of small size. Additionally, no information was available on spatiotemporal subject mobility, which could lead to exposure misclassification. However, people spend most of their time at home, and this is likely especially the case for pregnant women (Leech et al., 2002). Subjects may have moved home during the pregnancy period, which was not considered, and air pollution concentrations were averaged over 9 months, which could mask effects of variations in short-term exposure. Our analysis technique, CCA, is limited in that it assumes and only detects a linear intermodality relationship (Zhuang et al., 2020). Our study analysed brain structure at a single time-point in the neonatal period. Future studies using diffusion MRI are required to assess the relationship between exposure to air pollution and brain microstructure, and longitudinal MRI studies are required to assess the effects of air pollution exposure on brain development from the neonatal period to childhood.

## 5 Conclusions

This study shows for the first time in humans that prenatal air pollution exposure affects neonatal brain morphology, particularly relative cerebellar and ventricle size. The brain is especially vulnerable during this critical period of development and altered brain development in utero may have adverse consequences throughout the life course. This finding is an important addition to the growing body of evidence that gestational exposure to pollution affects health in early life and shows that reducing particulate matter concentrations and exposure during fetal life should be a public health priority. Our finding that lower NO_2_ exposure is related to larger relative ventricular and cerebellar size is more difficult to interpret, and requires further study.

### CRediT authorship contribution statement

**Brendan Bos:** Conceptualization, Methodology, Formal analysis, Writing – original draft, Writing – review & editing. **Ben Barratt:** Methodology, Writing – review & editing, Supervision, Project administration. **Dafnis Batalle:** Methodology, Formal analysis, Writing – review & editing. **Oliver Gale-Grant:** Methodology, Formal analysis, Writing – review & editing. **Emer J. Hughes:** Investigation, Data curation. **Sean Beevers:** Software. **Lucilio Cordero-Grande:** Software, Writing – review & editing. **Jana Hutter:** Methodology, Writing – review & editing. **Joseph V. Hajnal:** Funding acquisition, Methodology. **Frank J. Kelly:** Writing – review & editing. **A. David Edwards:** Funding acquisition, Writing – review & editing. **Serena J. Counsell:** Conceptualization, Methodology, Resources, Data curation, Writing – review & editing, Supervision, Project administration, Funding acquisition.

## Declaration of Competing Interest

The authors declare that they have no known competing financial interests or personal relationships that could have appeared to influence the work reported in this paper.

## Data Availability

Data from the developing human connectome project (dHCP) are available at https://biomedia.github.io/dHCP-release-notes/.
