# Supplementary material for: Prenatal exposure to air pollution is associated with structural changes in the neonatal brain
Source: Environ Int. 2023 Apr;174:107921. doi: 10.1016/j.envint.2023.107921 (PMC10410199; doi:10.1016/j.envint.2023.107921)
Supplement: Supplementary data 1 [file mmc1.docx]

**Supplementary Material**

**Prenatal exposure to air pollution is associated with structural changes in the neonatal brain**

Brendan Bos, Ben Barratt, Dafnis Batalle, Oliver Gale-Grant, Emer J. Hughes, Sean Beevers, Lucilio Cordero-Grande, Anthony N. Price, Jana Hutter, Joseph V. Hajnal, Frank J. Kelly, A. David Edwards, Serena J. Counsell

**Table of contents**

**1. Supplementary Methods**

**2. Supplementary Results**

**Table S1.** Single pollutant linear regression results for each relative brain volume and pollutant, first trimester.

**Table S2.** Single pollutant linear regression results for each relative brain volume and pollutant, second trimester.

**Table S3.** Single pollutant linear regression results for each relative brain volume and pollutant, third trimester.

**Figure S1.** Average annual concentrations of NO2 in µg/m^3^ in Greater London, 2016.

**Figure S2.** Average annual concentrations of PM10 in µg/m^3^ in Greater London, 2016.

**Figure S3.** A segmented image in a neonate at 40 weeks PMA showing the analysed brain regions in the sagittal, axial, and coronal planes. Left (L) to right (R), superior (S) to inferior (I), anterior (A) to posterior (P), cortical grey matter (dark blue), white matter (light brown), deep grey matter (brown), extracerebral CSF (beige), cerebellum (purple), brainstem (turquoise), amygdala and hippocampus (red), and ventricles (light green).

**1. Supplementary Methods.**

We examined sex-specific associations between prenatal exposure to air pollution and neonatal brain morphology. To this end we separated the study sample into male and female and conducted CCA analysis as outlined in the main manuscript.

We also investigated the correlations between exposures by trimester using Spearman’s rank correlation.

**2. Supplementary Results**

**2.1 Sex-specific associations**

The CCA of sex-specific associations identified three modes, and none of these exceeded the 95^th^ percentile of random permutations.

**2.2 Air pollution exposures by trimester**

Correlation between exposures by trimester was tested using Spearman’s rank correlation coefficient. Concentrations of air pollution by trimester were not correlated to each other (r < 0.1).

Table S1: Single pollutant linear regression results for each relative brain volume and pollutant, first trimester.

| Brain region (relative) | Pollutant | beta | p-value | p-FDR |
| --- | --- | --- | --- | --- |
| White Matter | NO_2_ | -0.017 | 0.578 | 0.918 |
|  | PM_2.5_ | 0.006 | 0.803 | 0.918 |
|  | PM_10_ | -0.009 | 0.724 | 0.918 |
| Grey Matter | NO_2_ | 0.021 | 0.468 | 0.918 |
|  | PM_2.5_ | -0.011 | 0.66 | 0.918 |
|  | PM_10_ | 0.0074 | 0.779 | 0.918 |
| Cerebellum | NO_2_ | -0.039 | 0.391 | 0.918 |
|  | PM_2.5_ | 0.014 | 0.73 | 0.918 |
|  | PM_10_ | 0.0167 | 0.698 | 0.918 |
| Brainstem | NO_2_ | 0.0474 | 0.326 | 0.869 |
|  | PM_2.5_ | 0.065 | 0.138 | 0.662 |
|  | PM_10_ | 0.045 | 0.320 | 0.869 |
| Ventricle | NO_2_ | -0.001 | 0.977 | 0.977 |
|  | PM_2.5_ | 0.132 | 0.01 | 0.12 |
|  | PM_10_ | 0.145 | 0.006 | 0.12 |
| Deep Grey Nuclei | NO_2_ | 0.016 | 0.744 | 0.918 |
|  | PM_2.5_ | -0.012 | 0.789 | 0.918 |
|  | PM_10_ | -0.033 | 0.50 | 0.918 |
| Extracerebral CSF | NO_2_ | 0.073 | 0.194 | 0.776 |
|  | PM_2.5_ | 0.095 | 0.064 | 0.512 |
|  | PM_10_ | 0.084 | 0.114 | 0.662 |
| Amygdala & Hippocampus | NO_2_ | -0.0066 | 0.902 | 0.941 |
|  | PM_2.5_ | -0.008 | 0.855 | 0.933 |
|  | PM_10_ | -0.058 | 0.246 | 0.843 |

Table S2: Single pollutant linear regression results for each relative brain volume and pollutant, second trimester.

| Brain region (relative) | Pollutant | beta | p-value | p-FDR |
| --- | --- | --- | --- | --- |
| White Matter | NO_2_ | -0.02 | 0.512 | 0.829 |
|  | PM_2.5_ | -0.03 | 0.276 | 0.829 |
|  | PM_10_ | -0.026 | 0.368 | 0.829 |
| Grey Matter | NO_2_ | 0.027 | 0.342 | 0.829 |
|  | PM_2.5_ | 0.019 | 0.458 | 0.829 |
|  | PM_10_ | 0.0143 | 0.606 | 0.829 |
| Cerebellum | NO_2_ | -0.043 | 0.363 | 0.829 |
|  | PM_2.5_ | 0.005 | 0.889 | 0.915 |
|  | PM_10_ | 0.004 | 0.915 | 0.915 |
| Brainstem | NO_2_ | 0.024 | 0.622 | 0.829 |
|  | PM_2.5_ | 0.023 | 0.601 | 0.829 |
|  | PM_10_ | 0.048 | 0.303 | 0.829 |
| Ventricle | NO_2_ | -0.1 | 0.086 | 0.829 |
|  | PM_2.5_ | -0.041 | 0.427 | 0.829 |
|  | PM_10_ | -0.009 | 0.864 | 0.915 |
| Deep Grey Nuclei | NO_2_ | 0.006 | 0.906 | 0.915 |
|  | PM_2.5_ | 0.033 | 0.492 | 0.829 |
|  | PM_10_ | 0.037 | 0.458 | 0.829 |
| Extracerebral CSF | NO_2_ | 0.021 | 0.721 | 0.911 |
|  | PM_2.5_ | 0.042 | 0.418 | 0.829 |
|  | PM_10_ | 0.081 | 0.144 | 0.829 |
| Amygdala & Hippocampus | NO_2_ | -0.011 | 0.841 | 0.915 |
|  | PM_2.5_ | -0.028 | 0.572 | 0.829 |
|  | PM_10_ | -0.042 | 0.423 | 0.829 |

Table S3: Single pollutant linear regression results for each relative brain volume and pollutant, third trimester.

| Brain region (relative) | Pollutant | beta | p-value | p-FDR |
| --- | --- | --- | --- | --- |
| White Matter | NO_2_ | -0.013 | 0.658 | 0.969 |
|  | PM_2.5_ | -0.005 | 0.844 | 0.969 |
|  | PM_10_ | -0.007 | 0.812 | 0.969 |
| Grey Matter | NO_2_ | 0.024 | 0.39 | 0.969 |
|  | PM_2.5_ | 0.006 | 0.813 | 0.969 |
|  | PM_10_ | 0.011 | 0.674 | 0.969 |
| Cerebellum | NO_2_ | -0.059 | 0.19 | 0.969 |
|  | PM_2.5_ | 0.022 | 0.60 | 0.969 |
|  | PM_10_ | -0.032 | 0.472 | 0.969 |
| Brainstem | NO_2_ | 0.054 | 0.256 | 0.969 |
|  | PM_2.5_ | 0.033 | 0.462 | 0.969 |
|  | PM_10_ | 0.038 | 0.424 | 0.969 |
| Ventricle | NO_2_ | -0.057 | 0.308 | 0.969 |
|  | PM_2.5_ | 0.002 | 0.969 | 0.969 |
|  | PM_10_ | 0.002 | 0.963 | 0.969 |
| Deep Grey Nuclei | NO_2_ | 0.006 | 0.902 | 0.969 |
|  | PM_2.5_ | 0.019 | 0.691 | 0.969 |
|  | PM_10_ | 0.009 | 0.860 | 0.969 |
| Extracerebral CSF | NO_2_ | 0.038 | 0.492 | 0.969 |
|  | PM_2.5_ | 0.035 | 0.508 | 0.969 |
|  | PM_10_ | 0.015 | 0.782 | 0.969 |
| Amygdala & Hippocampus | NO_2_ | 0.204 | 0.067 | 0.969 |
|  | PM_2.5_ | 0.043 | 0.389 | 0.969 |
|  | PM_10_ | 0.062 | 0.243 | 0.969 |


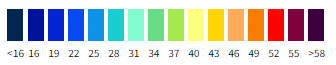

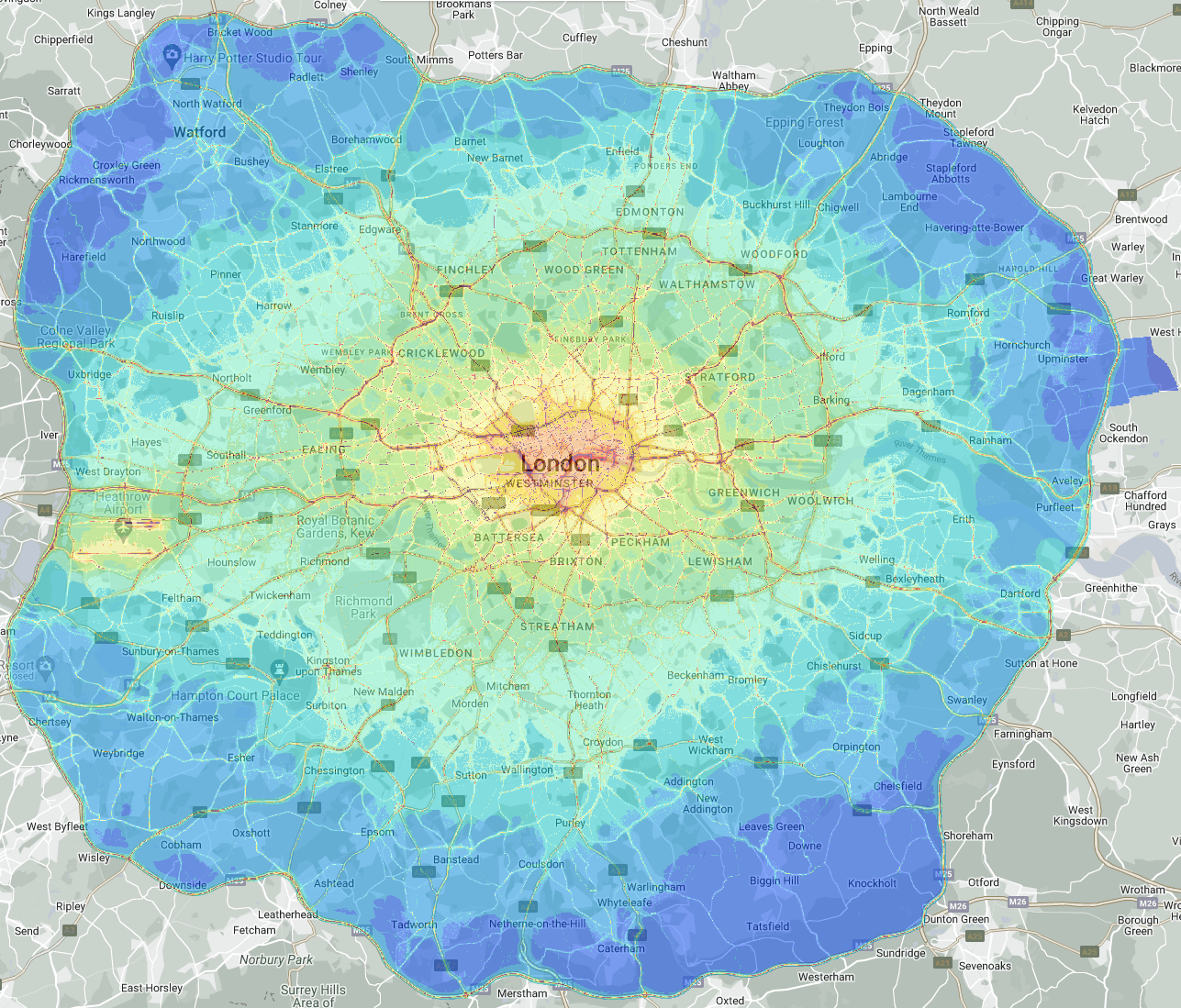


Figure S1: Average annual concentrations of NO_2_ in µg/m^3^ in Greater London, 2016.


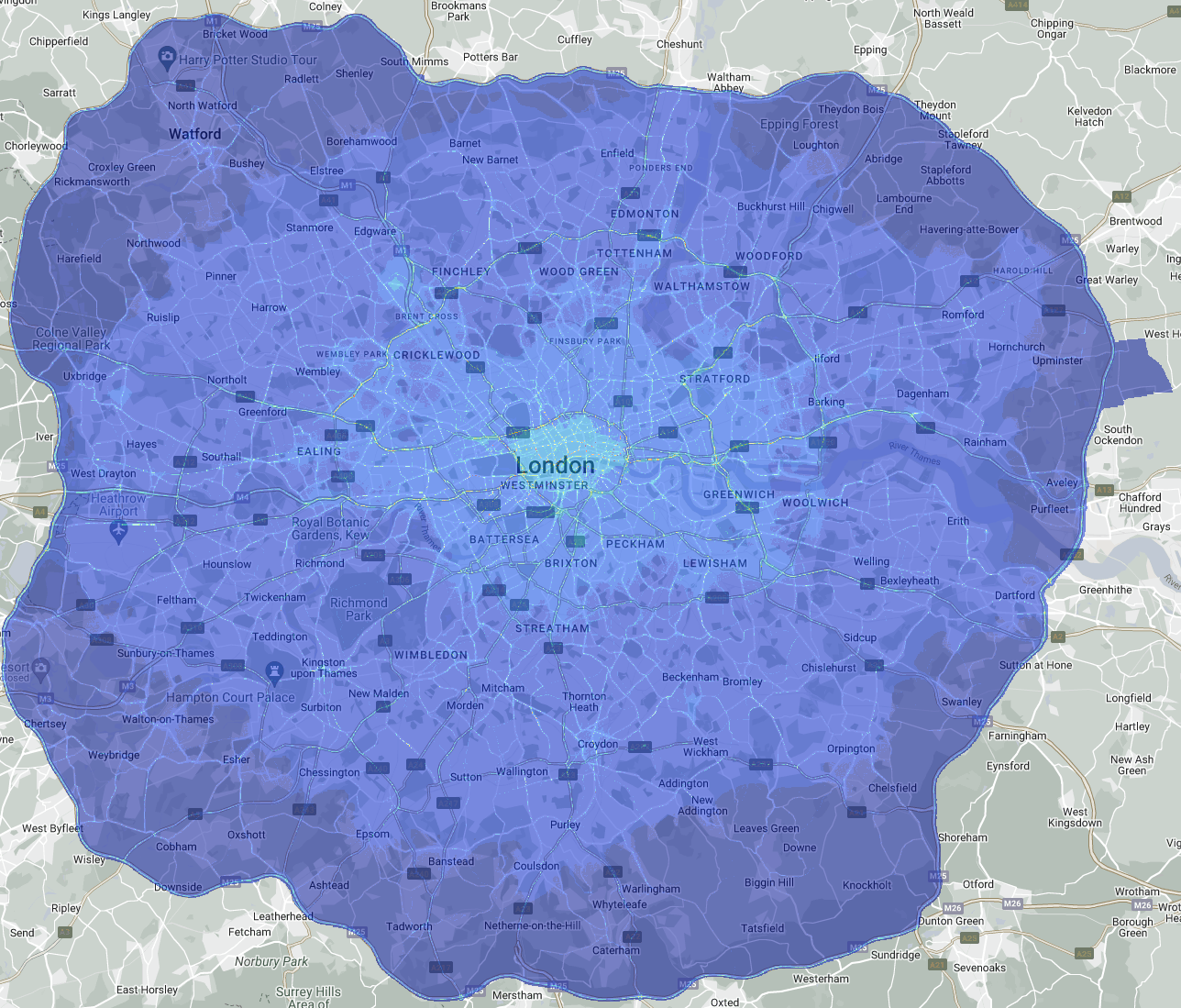

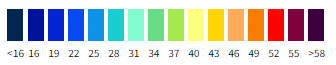


Figure S2: Average annual concentrations of PM_10_ in µg/m^3^ in Greater London, 2016.


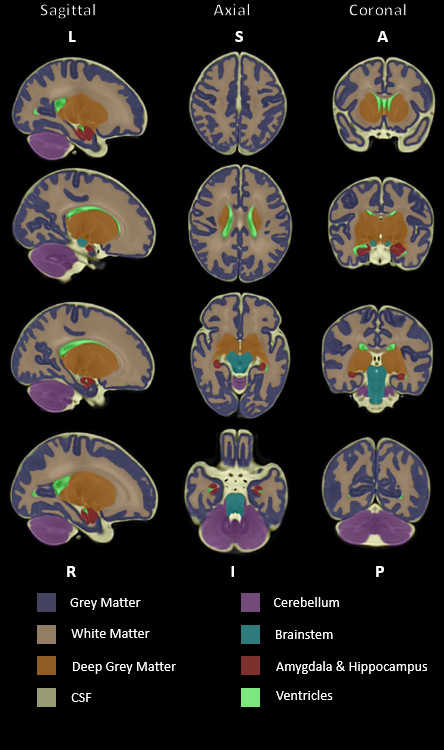


Figure S3: A segmented image in a neonate at 40 weeks PMA showing the analysed brain regions in the sagittal, axial, and coronal planes. Left (L) to right (R), superior (S) to inferior (I), anterior (A) to posterior (P), cortical grey matter (dark blue), white matter (light brown), deep grey matter (brown), extracerebral CSF (beige), cerebellum (purple), brainstem (turquoise), amygdala and hippocampus (red), and ventricles (light green).
